# CircNFIC Balances Inflammation and Apoptosis by Sponging miR-30e-3p and Regulating DENND1B Expression

**DOI:** 10.3390/genes12111829

**Published:** 2021-11-19

**Authors:** Yangfeng Chen, Zhijun Wang, Xiaolan Chen, Xi Peng, Qinghua Nie

**Affiliations:** 1 Guangdong Laboratory for Lingnan Modern Agricultural Science and Technology, South China Agricultural University, Guangzhou 510642, China; yangfengchen1213@163.com (Y.C.); zhijunwang@stu.scau.edu.cn (Z.W.); 2 Guangdong Provincial Key Lab of Agro-Animal Genomics and Molecular Breeding and Key Lab of Chicken Genetics, Breeding and Reproduction, Ministry of Agriculture, Guangzhou 510642, China; 3 College of Animal Science, South China Agricultural University, Guangzhou 510642, China; 18123025248@163.com; 4 School of Life Sciences, Chongqing University, Chongqing 401331, China; xiaolanchend@163.com

**Keywords:** inflammation, apoptosis, circular RNA, miR-30e-3p/DENND1B axis, Lipopolysaccharide

## Abstract

Disordered inflammation and apoptosis are closely related to diseases, and inflammation can also promote cell apoptosis, where growing evidence has shown that circular RNAs (circRNAs) play important roles. Lipopolysaccharide (LPS) is the main component of the cytoderm of gram-negative bacterium, which can cause inflammatory responses in macrophages. We constructed an inflammatory model by exposing chicken macrophage cell lines (also known as HD11) to LPS for in vitro experiments. In this study, we validated a novel circRNA—circNFIC—which was dramatically up-regulated in tissues infected by coccidia and cells exposed to LPS. Besides, circNFIC could significantly promote the expression levels of pro-inflammation factors, including (IL-1β, TNFα, and IFNγ) and pro-apoptosis maker genes (caspase 3 and caspase 8) in HD11 exposed to LPS or not. In terms of mechanism, circNFIC exerted notable effects on DENND1B to regulate cell inflammation and apoptosis by sponging miR-30e-3p. The molecular functions played by miR-30e-3p and DENND1B have been explored, respectively. In addition, the effects of circNFIC knockdown suppressing the expression of pro-inflammatory and pro-apoptosis functions could be reversed by a miR-30e-3p inhibitor. On the whole, circNFIC promoted cell inflammation and apoptosis via the miR-30e-3p/DENND1B axis.

## 1. Introduction

The poultry industry plays an important role in reducing malnutrition, reducing poverty, and promoting economic growth, because produced chicken and eggs are important sources of animal protein, and may be the only source of high-quality protein for the poor [1]. However, the industry has been severely affected by coccidiosis for a long time, causing huge economic losses [2]. Some researchers updated the Williams model and estimated that the global poultry industry’s loss due to chicken coccidiosis was about 10.4 billion pounds in 2016 [3,4]. Coccidiosis invades the intestines of poultry [5], activates cellular immunity and humoral immunity in the host poultry [6], and includes mediating inflammation by affecting the transcription of cytokines such as TNFα, IL-1β, and IFN-γ [7,8]. The current prevention and control measures for coccidiosis mainly consist of vaccination and coccidiosis inhibitor drugs, but the effect is not significant [9] because they did not affect a radical cure [10,11]. Due to the complexity of vaccine production capacity, cost, and cross-protection, and the continuous emergence of drug-resistant coccidia [12,13], there is an urgent need for more novel and efficient strategies against coccidiosis.

Circular RNAs (circRNAs) are non-coding RNA molecules that are widely expressed in eukaryotes [14]. The post-transcriptional modification of circRNA undergoes a “back-splicing” process that is different from linear transcripts after transcription from pre-mRNA and is expressed both inside and outside the nucleus [15] so that the downstream splice donor site is covalently linked to the upstream splice acceptor site [16]. Due to its special ring structure, circRNA has strong resistance to RNase R digestion and inherit conservation [14,16,17], which can also be used as new biomarkers of some diseases. Since 2011, Salmena proposed the ceRNA hypothesis [18], where an increasing number of circRNAs have been found to act as sponges, playing a regulator role [19,20,21], which consist of one or multiple exons [22]. Some studies found that circRNAs played an important role in the polarization and apoptosis of macrophages [23,24] and regulated the inflammation response of macrophages exposed to lipopolysaccharide (LPS) [25,26]. Therefore, we know that circRNA has great potential in the process of apoptosis and inflammation. Our previous uploaded a comprehensive expression profile of circRNA, in which gga-circ_0002001 was significantly up-regulated in the expression of coccidia infected Sasso broilers (*p* < 0.05), and circ_0002001 was derived from the pre-mRNA of nuclear factor I C (NFIC), which involves inflammation and apoptosis [27,28], where we termed it circNFIC.

MiRNAs are small non-coding RNAs molecules with a length of 19–25 nucleotides [29], which can specifically bind to the 3′ untranslated region (UTR) of the target mRNA through the 2–7 nucleotides of the 5′ end by complementary base pairing [30], thereby regulating target genes expression post-transcriptionally [29]. It was reported that miR-30e-3p plays an important role in promoting autophagy and inhibiting apoptosis of cardiomyocytes in mice during ischemia or hypoxia [31]. Another study found that miR-30e-3p is associated with immunological responses and vascular remodeling [32]. Therefore, we want to explore whether miR-30e-3p can perform similar functions in chickens.

The proteins of the DENND1A-C (also known as connecdenn1-3) family can function as guanine exchange factors (GEF) and directly interact with Rab35 through the DENN domain [33,34], which is ubiquitously expressed and evolutionarily conserved in eukaryotes [34]; thereby, it regulates a variety of cellular functions, including transmembrane transport and intercellular signal transmission. Among these functions, DENND1B has been reported to be involved in the occurrence of immune response and a variety of diseases, its expression disorder is associated with inflammatory bowel disease (IBD) [35] and asthma [36,37], and it can regulate T cell receptor signaling, which is related to the pathogenesis of asthma [38]. In addition, DENND1B might regulate TNFR1-mediated apoptosis in neuronal cells [39]. We pay particular attention to the functions of DENND1B in inflammatory response and apoptosis, as well as its upstream regulatory mechanism.

## 2. Materials and Methods

### 2.1. Cell Culture and Transfection

The chicken macrophage cell lines (HD11) were cultured in Roswell Park Memorial Institute (RPMI) Medium 1640 (Gibco, Carlsbad, CA, USA) with 10% fetal bovine serum (Gibco) and 1% penicillin/streptomycin (Invitrogen, Carlsbad, CA, USA). The chicken fibroblast cell lines (DF-1) were cultured in DMEM (Gibco) with 10% fetal bovine serum and 1% penicillin/streptomycin (Invitrogen). All the cells were cultured in the incubator with 5% CO2 at 37 °C. Before transfections, HD11 was blanked into culture plates, with a number of 5 × 10^5^ for a 6-well culture plate, 1 × 10^5^ for a 12-well culture plate, 5 × 10^4^ for a 24-well culture plate, and 1 × 10^4^ for a 96-well culture plate. Transfections were performed with Lipofectamine 3000 reagent (Invitrogen) according to the instructions, when the cells grew to 60–80% confluence, with at least three independent technical replications.

The HD11 cell lines were obtained from Prof. Susan J. Lamont (Department of Animal Science, Iowa State University, Ames, IA, USA) and Prof. Guobin Chang (Key Laboratory of Animal Genetics and Breeding and Molecular Design of Jiangsu Province, Yangzhou University, Yangzhou, China).

### 2.2. Construction of Cell Inflammatory Model

Lipopolysaccharide (LPS) (Solarbio, Beijing, China) was used to induce inflammation and apoptosis in HD11. HD11 were exposed to LPS of the concentration at 1 µg/mL after passage for 4 h, and the control group was treated with phosphate buffer solution (PBS) (Gibco) instead of LPS. The pro-inflammatory factors (IL-1β, TNFα), pro-apoptosis maker genes (caspase 3 and caspase 8), and inducible nitric oxide synthase (iNOS) were detected by qRT-PCR after culturing for 12 h, which indicated whether the inflammatory model was successfully constructed (Figure S1).

### 2.3. Circular Structure Confirmation

PCR with divergent primer, Sanger sequencing, and RNase R treatment assay were performed to confirm the circNFIC. The back-spliced junction (BSJ) of circNFIC was amplified by a divergent primer, delivered to Tsingke (Beijing, China) for Sanger sequencing, and blasted with the back-spliced region by DNAstar software (https://www.dnastar.com/, accessed on 4 October 2020). The RNase R treatment assay was performed according to the instrument of the Ribonuclease R kit (Geneseed, Guangzhou, China). The primers are listed in Table S1.

### 2.4. RNA Oligonucleotides used in This Study

All the small interfering RNA (siRNA), including si-circNFIC-1, si-circNFIC-2, si- DENND1B-1, si-DENND1B-2, si-DENND1B-3, siRNA negative control (si NC), and mimics, including miR-30e-3p mimic, mimic negative control (mimic NC), miR-30e-3p inhibitor, and inhibitor negative control (inhibitor NC), were designed and synthesized by RiboBio (Guangzhou, China) and the sequences are shown in Table S2.

### 2.5. Construction of Vectors

The primers, overexpression vectors—including overexpression of circNFIC and overexpression of DENND1B—and pmirGLO (pGLO) dual-luciferase reporter vectors, including pGLO-circNFIC-WT, pGLO-circNFIC-MUT, pGLO-DENND1B-WT1, pGLO-DENN1B-MUT1, pGLO-DENND1B-WT2, pGLO-DENN1B-MUT2, pGLO-DENND1B-WT3, and pGLO-DENN1B-MUT3, in this study were purchased from Tsingke (Beijing, China). Primers used for vector construction are listed in Table S2.

### 2.6. miRNA Targets Prediction and RNAhybrid Detection

MiRDB (http://mirdb.org/, accessed on 1 August 2021) and RNAhybrid (https://bibiserv.cebitec.uni-bielefeld.de/rnahybrid?id=rnahybrid_view_submission, accessed on 1 August 2021) were used to predict target gene of miR-30e-3p and calculate the combined minimum free energy (MFE) between miR-30e-3p and circNFIC or DENND1B 3’ untranslated regions (UTR), respectively.

### 2.7. Biotin Labeled Probe Pull-Down Assay

The biotin labeled miR-30e-3p probe was designed and synthesized by Biosense (Guangzhou, China). The pull-down assay was performed in HD11 according to the instrument of the microRNA pull-down kit (Biosense).

### 2.8. RNA Fluorescent In Situ Hybridization

The RNA fluorescent in situ hybridization (FISH) assays were carried out to detect the cellular location of circNFIC and the co-localization with miR-30e-3p in HD11 with a FISH kit (RiboBio). CY3-labeled circNFIC probes were designed and synthesized by RiboBio, and the FAM-labeled miR-30e-3p probes were designed and synthesized by GenePharma (Shanghai, China). Briefly, HD11 was planked into a 3.5 cm culture dish with a number about 2 × 10^5^. HD11 was incubated with probes (10 µM) at 37 °C overnight after fixation and hybridization. Then, cell nuclei were stained with DAPI (Beyotime, Shanghai, China) for 10 min. Lastly, the images were captured by laser scanning confocal microscope (Leica, Wetzlar, Germany).

### 2.9. RNA Isolation, Reverse Transcription

Total RNA was isolated from tissues and cells following the standard protocol of the chloroform method. The cells were incubated with 0.8 mL RNAiso Plus (Takara, Shiga Japan) for 5 min at room temperature and then shaken for 15 s. Added 0.2 mL chloroform (Damao, Tianjin, China), and incubated for 5 min at room temperature, and the cell lysis mix was centrifuged for 15 min at 12,000× *g*, 4 °C. After that, 0.5 mL supernatant was transferred into a new nuclease-free tube, and 0.5 mL isopropanol (Damao) was added to the above-mentioned supernatant. The mixture was shaken by hand for 15 s and incubated for 30 min at −80 °C, and centrifugation was performed for 10 min at 12,000× *g*, 4 °C. Then, we discarded the supernatant and washed the tube with 75% ethanol (Damao). The RNA was centrifuged for 5 min at 12,000× *g*, 4 °C. The supernatant was also discarded, and the tube was diluted with 30 µL nuclease-free water (TransGen, Beijing, China) after drying flat for 5 min at room temperature. The concentration and quality of RNA were determined using a NanoDrop One spectrophotometer (ThermoFisher, Waltham, MA USA). Then, the RNA sample was placed at −80 °C for use later.

Complicated DNA (cDNA) was synthesized from mRNA and miRNA, according to the instrument of the RT kit with gDNA cleaning for qPCR (AccurateBio, Shenzhen, China) and Frist strand synthesis kit (Toyobo, Shanghai, China), respectively, which includes the gDNA cleaning step and synthesis step. Additionally, both RNA isolation and reverse transcription had at least three independent technical replications, just the same as transfection.

### 2.10. Quantitative Real-Time PCR and Western Blotting

The quantitative real-time PCR (qRT-PCR) assays were performed with ChamQ SYBR qPCR Master Mix (Vazyme, Nanjing, China) in the 96-well PCR plates (Monad, Wuhan, China). The primers used for qRT-PCR are listed in Table S2. Cellular proteins were extracted by ice-cold radio immunoprecipitation assay (RIPA, Beyotime) buffer with 1% phenylmethanesulfonyl fluoride (PMSF, Beyotime) protease inhibitor, mixed with proteins loading buffer (TransGen). The assays were performed as previously reported [40]. The primary antibody, including polyclonal rabbit anti-DENND1B antibody (LifespanBio, Seattle, WA, USA, 1:500), anti-GAPDH (Abbkine, Wuhan, China, 1:5000), as well as the second antibody, including HRP conjugated goat anti-rabbit IgG (Abbkine, 1:10,000) and HRP conjugated goat anti-mouse IgG (Abbkine, 1:10,000), were used for probing in this study. The BeyoRCL Star Kit (Beyotime) was used for color rendering in the Odyssey Fc Image System (LI-COR, Lincoln, NE, USA). Furthermore, both qRT-PCR and western blot had at least three independent technical replications, just the same as transfection.

### 2.11. Dual-Luciferase Reporter Assay

Firefly and renilla luciferase activities were detected according to the manufacturer’s instructions of the Dual-GLO Luciferase Assay System Kit (Promega, Madison, WI, USA), after co-transfection of pmirGLO vectors mentioned above with miR-30e-3p mimic or mimic NC, respectively, for 48 h, with eight independent replications, detected by Multi-function microplate reader (Biotek, Winooski, VT, USA).

### 2.12. Statistical Analysis

All data was shown as mean ± standard error mean (SEM), with at least three independent replications. The statistically significant difference between groups was assessed by Student’s *t*-test. We considered *p* < 0.05 to be statistically significant. * *p* < 0.05; ** *p* < 0.01; *** *p* < 0.001, ns—no significance.

## 3. Results

### 3.1. Characteristics and Subcellular Localization of circNFIC

**Figure 1:**
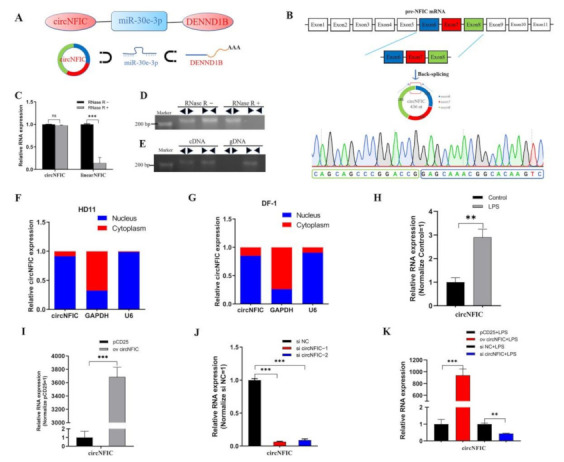
Characteristics and subcellular localization of circNFIC. (**A**) The differently expressed regulation interaction network (up-regulation and down-regulation transcripts were highlighted in red or blue, respectively). (**B**) In the formation of circNFIC, the BSJ was amplified and validated by divergent primers and sanger sequencing. (**C**,**D**) The resistance of circNFIC was detected by the RNase R digestion assay. (**E**) Divergent primers amplified circACSL1 from cDNA, but not from gDNA. (**F**,**G**) The separation of nuclear and cytoplasmic was performed in HD11 cells and DF-1 cells. (**H**) The expression of circNFIC was induced by LPS stimulation. (**I**,**J**) The efficiency of overexpression and knockdown of circNFIC was detected by qRT-PCR. (**K**) The efficiency of overexpression and knockdown of circNFIC was detected by qRT-PCR during LPS stimulation. Data are presented as mean ± SEM (*n* ≥ 3 biologically independent samples). ** *p* < 0.01; *** *p* < 0.001 (Student’s *t*-test).

In our unpublished sequencing data, joint analyses of differentially expressed (DE) circRNA, miRNA, and mRNA were performed, and a differently expressed regulation interaction network was screened out, namely the circNFIC-miR-30e-3p-DENND1B interaction network (Figure 1A). Among them, circNFIC and DENND1B were differentially up-regulated, and miR-30e-3p was differentially down-regulated. CircNFIC was derived from exon 6, exon 7, and exon 8 of nuclear factor I C (NFIC) pre-mRNA (Figure 1B), which can suppress LPS-initiated innate immune responses [41]. Firstly, the divergent primers were designed to amplify the back-spliced junction (BSJ), and the products were delivered to Qsingke (Beijing, China) for Sanger sequencing (Figure 1B). Then, the RNase R digestion assay was carried out to digest the linear RNAs, and the expression of linear NFIC mRNA decreased significantly compared with circNFIC (Figure 1C,D). After that, the divergent primers and convergent primers were used to amplify NFIC and circNFIC, and the gDNA and cDNA served as the templates (Figure 1E), respectively. The separations of nuclear and cytoplasmic were performed in HD11 cells and DF-1 cells to detect the subcellular localization of circNFIC, both of them revealed that the localization of circNFIC was both in the cytoplasm and nucleus (Figure 1F,G). Further, fluorescence in situ hybridization (FISH) was carried out also to confirm this (Figure S4A). Some researchers have shown that the macrophage migration inhibitory factor (MIF) could enhance innate immune responses during coccidia infection [8], as the MIF promotes the transcription of TNFα, IL-1β, and IFNγ [42,43]. At the same time, the coccidia induced terrible intestinal inflammation [44]. The different expressions of circNFIC in chickens infected by coccidia led us to consider whether it is related to inflammation and apoptosis. To investigate the role that circNFIC plays, we constructed an inflammation model by exposing LPS to HD11 or not to HD11, to simulate inflammation responses and normal responses, respectively. Subsequently, we assessed the level of circNFIC after exposing HD11 cells to LPS or not, and afterwards, we found the expression of circNFIC was significantly up-regulated in LPS-reduced HD11, compared with the mock group (Figure 1H), which suggested that circNFIC might play a positive role in inflammation and apoptosis. Then, the overexpression vector and siRNA were designed and constructed, and we confected them into normal HD11 to detect the efficiency of overexpression and knockdown (Figure 1I,J), in the same way as the HD11 was exposed to LPS (Figure 1K).

### 3.2. circNFIC Aggravates Inflammation, Apoptosis, and Direct Targets miR-30e-3p

**Figure 2:**
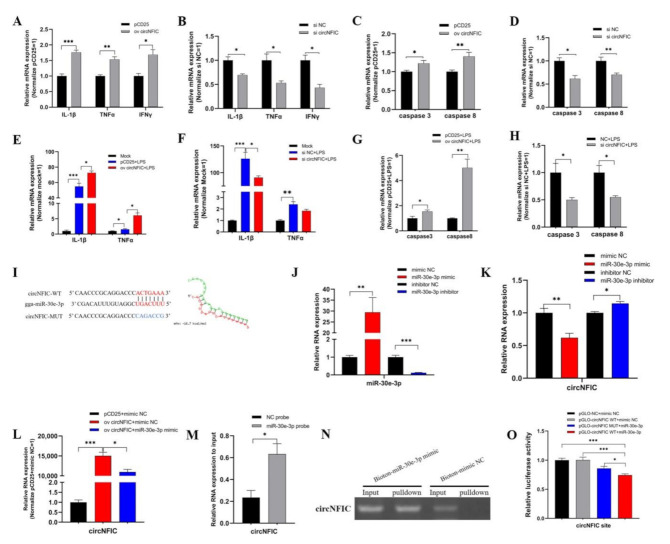
CircNFIC aggravates inflammation and apoptosis, and directly targets miR-30e-3p. (**A**,**B**) The qRT-PCR assays showed that the mRNA expression levels of IL-1β, TNFα, and IFNγ after transfecting overexpression vector and siRNA of circNFIC in HD11. (**C**,**D**) The expression levels of caspase 3 and caspase 8 with overexpression and knockdown of circNFIC in HD11. (**E**,**F**) The levels of IL-1β, TNFα, and IFNγ with overexpression and knockdown of circNFIC in HD11 with LPS stimulation. (**G**,**H**) After overexpression and knockdown of circNFIC, the levels of caspase 3 and caspase 8 in HD11 with LPS stimulation. (**I**) The potential binding site sequence (were highlighted in red) of miR-30e-3p on circNFIC, and the interaction model between miR-30e-3p and circNFIC, by RNAhybrid software. (**J**) The efficiency of overexpression and knockdown of miR-30e-3p was detected by qRT-PCR. (**K**,**L**) The expression of circNFIC was suppressed by miR-30e-3p. (**M**) The enrichment of circNFIC in the biotinylated miR-30e-3p mimics pull-down RNA. (**N**) The qRT-PCR products of circNFIC in RNA pull-down. (**O**) Dual-luciferase assay verified the binding relationship between circNFIC and miR-30e-3p. Data are presented as mean ± SEM (*n* ≥ 3 biologically independent samples). * *p* < 0.05; ** *p* < 0.01; *** *p* < 0.001 (Student’s *t*-test).

To explore the specific role of circNFIC, we overexpressed circNFIC in HD11; consequently, we found the significant up-regulation of pro-inflammatory factors, including IL-1β, TNFα, and IFNγ (Figure 2A), as well as caspase 3 and caspase 8 (Figure 2C)—the key factors in apoptosis—and when we carried out knockdown on circNFIC, these pro-inflammatory factors—caspase 3 and caspase 8—presented an opposite trend (Figure 2B,D). Further, we transfected overexpression vectors of circNFIC into HD11, and, when exposing it to LPS stimulating, we found the expressions of IL-1β and TNFα were further promoted, based on dramatically increased stimulated by LPS (Figure 2E). Whereas the knockdown of circNFIC resulted in the low-expression of IL-1β and TNFα compared with the levels induced by LPS stimulation (Figure 2F). As for caspase 3 and caspase 8, the overexpression and knockdown of circNFIC presented significant up-regulation or down-regulation under LPS stimulation, respectively (Figure 2G,H). For now, we deduced that circNFIC could exert pro-inflammatory and pro-apoptotic effects in HD11.

To obtain the mechanism of the role circNFIC plays, we used the miRNA target prediction software (https://bibiserv.cebitec.uni-bielefeld.de/, accessed on 1 August 2021) and found that miR-30e-3p matched with one site in circNFIC (Figure 2I) and the RNA duplex’s combined minimum free energy (MFE) was approximately −16.7 kcal/mol, which indicated the interaction was more likely. Then the mimic and inhibitor of miR-30e-3p were confected into HD11 to detect the efficiency of overexpression and knockdown, respectively (Figure 2J). At the same time, we found the expression of circNFIC significantly down-regulated after miR-30e-3p overexpressed and that miR-30e-3p knockdown can induce an opposite trend (Figure 2K). Additionally, the level of circNFIC was also suppressed by miR-30e-3p mimic, based on circNFIC overexpression (Figure 2L), which further indicated that miR-30e-3p could cause the suppression of expression of circNFIC. The RNA pull-down assay was carried out in HD11 to verify the physical interaction between circNFIC and miR-30e-3p, and qRT-PCR showed that the enrichment of circNFIC in the biotinylated miR-30e-3p mimic group was significantly more than bio-NC group (Figure 2M); the agarose gel electrophoresis of qRT-PCR products also confirmed this (Figure 2N). Notably, the fragments of circNFIC contained wild-type or mutant miR-30e-3p binding sites, which were synthesized and inserted into dual-luciferase reporter vectors (Figure 2I). The luciferase activity of the pGLO-circNFIC+miR-30e-3p group was dramatically reduced compared with the pGLO-NC and mimic NC group, the pGLO-circNFIC and mimic NC group, as well as pGLO-circNFIC+miR-30e-3p group (Figure 2O). These results further validated the binding relationship between circNFIC and miR-30e-3p. Afterwards, we found circNFIC and miR-30e-3p were subcellular co-localized by RNA-FISH assay (Figure S4B).Taken together, circNFIC was the direct target of miR-30e-3p and could be regulated by miR-30e-3p to function, but the downstream processes remain unknown for now.

### 3.3. The gga-miR-30e-3p Suppresses Inflammation and Apoptosis and Direct Targets DENND1B

**Figure 3:**
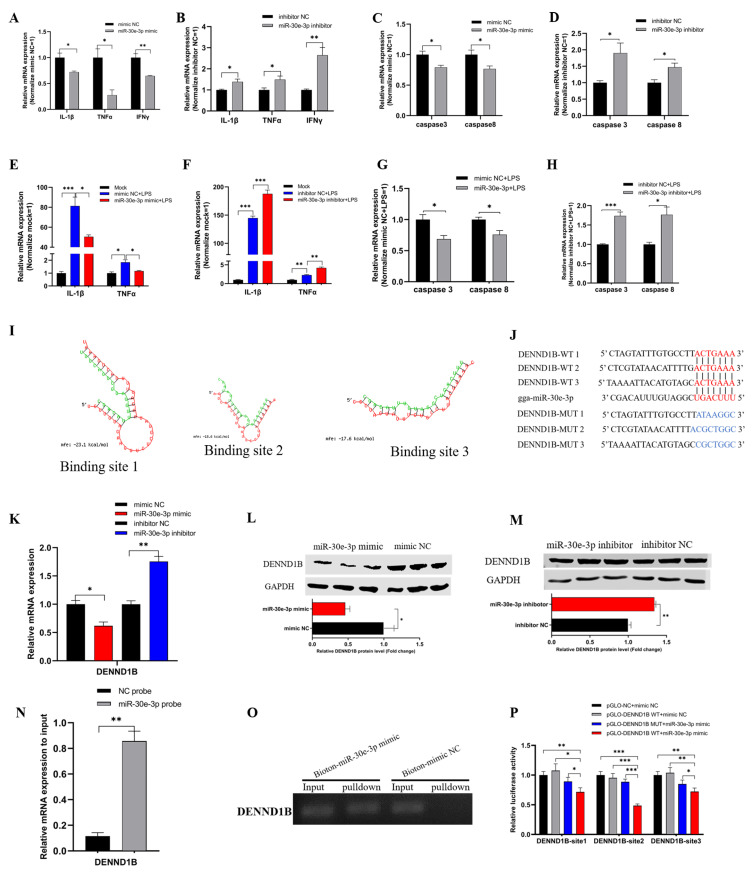
The gga-miR-30e-3p suppresses inflammation and apoptosis. (**A**,**B**) Mir-30e-3p suppressed the mRNA expression levels of pro-inflammatory factors. (**C**,**D**) Mir-30e-3p suppressed the mRNA expression of pro-apoptosis maker genes. (**E**,**F**) The expressions of pro-inflammatory factors were down-regulated by miR-30e-3p in HD11 exposed to LPS. (**G**,**H**) The mRNA expressions of pro-apoptosis maker genes were down-regulated by miR-30e-3p in HD11 exposed to LPS. (**I**) MiR-30e-3p matched three binding sites in 3′ UTR of DENND1B. (**J**) The potential binding site sequence (were highlighted in red) of miR-30e-3p on 3′ UTR of DENND1B. (**K**,**M**) Mir-30e-3p suppressed both mRNA and protein expression levels of DENND1B. (**N**) The enrichment of DENND1B in the biotinylated miR-30e-3p mimics pull-down RNA. (**O**) The qRT-PCR products of DENND1B in pull-down RNA. (**P**) Dual-luciferase assay verified the binding relationship between DENND1B and miR-30e-3p. Data are presented as mean ± SEM (n ≥ 3 biologically independent samples). * *p* < 0.05; ** *p* < 0.01; *** *p* < 0.001 (Student’s *t*-test).

To explore the role of miR-30e-3p in inflammation and apoptosis of HD11, we firstly transfected miR-30e-3p mimic and inhibitor into HD11 to detect the mRNA expression levels of inflammatory marker genes, including IL-1β, TNFα, and IFNγ, as well as apoptosis marker genes, including caspase 3 and caspase 8, evaluated by qRT-PCR. Overexpression of miR-30e-3p significantly down-regulated the levels of IL-1β, TNFα, and IFNγ compared with the mimic NC group (Figure 3A). Conversely, the knockdown of miR-30e-3p notably induced the levels of IL-1β, TNFα, and IFNγ (Figure 3B). Consequently, the expression levels of caspase 3 and caspase 8 were observably suppressed by overexpression of miR-30e-3p (Figure 3C); whereas, the levels presented an opposite trend after knockdown of miR-30e-3p (Figure 3D). Next, we conducted the experiment in HD11, exposing to LPS. We noticed a significant reduction of IL-1β, TNFα, and IFNγ based on their dramatic increase from LPS stimulation (Figure 3E). Further, the miR-30e-3p inhibitor brought a notable promotion of IL-1β, TNFα, and IFNγ after LPS stimulation (Figure 3F). In addition, the mRNA expression levels of caspase 3 and caspase 8 could also be reduced by miR-30e-3p during stimulation by LPS (Figure 3G), while knockdown of miR-30e-3p promoted the expression of caspase 3 and caspase 8 (Figure 3H). In short, miR-30e-3p could suppress the mRNA expression of IL-1β, TNFα, and IFNγ, as well as caspase 3 and caspase 8 in HD11, when exposed to LPS or not.

For the purpose to explore the downstream processes about miR-30e-3p, we used the miRDB (http://mirdb.org/, accessed on 1 August 2021) to find the target gene of miR-30e-3p, and we found that miR-30e-3p matched three binding sites in 3′ untranslated regions (UTR) of DENND1B (Figure 3I,J). Then, we transfected miR-30e-3p mimic or inhibitor into HD11, and qRT-PCR and western blot were carried out to detect the mRNA and protein expression levels of DENND1B. The mRNA and protein levels of DENND1B were notably reduced after the overexpression of miR-30e-3p (Figure 3K,L), and the miR-30e-3p inhibitor brought significant up-regulation of mRNA and expression of DENND1B (Figure 3K,M). Subsequently, qRT-PCR showed that DENND1B was significantly pulled down by miR-30e-3p probes, compared with NC probes (Figure 3N). The agarose gel electrophoresis of qRT-PCR products also confirmed this (Figure 3O). Similarly, the fragments of 3′ UTR of DENND1B, which contained 3 wildtype miR-30e-3p binding sites, were synthesized and inserted into pmirGLO vector (Figure 3J), all of the luciferases of these wildtype vectors and the miR-30e-3p mimic group were dramatically reduced compared with the corresponding wildtype vectors and mimic NC, as well as NC vector and mimic NC (Figure 3P). In brief, these results indicated that the binding relationship between miR-30e-3p and DENND1B was the most stable format.

### 3.4. circNFIC Served as a Sponge of miR-30e-3p to Regulate the Expression of DENND1B

**Figure 4:**
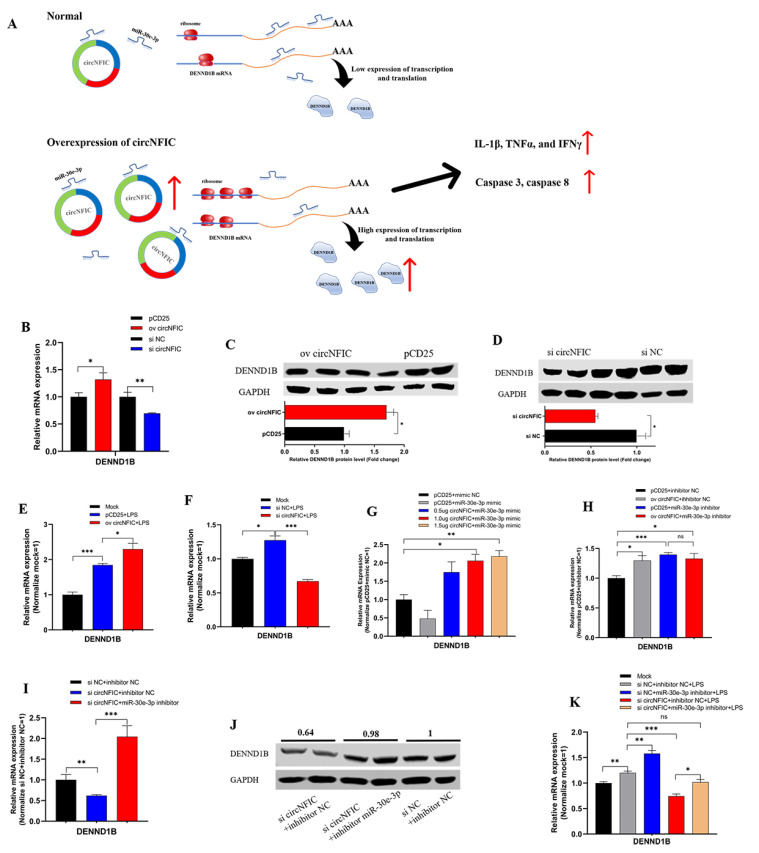
CircNFIC served as a sponge of miR-30e-3p to regulate the expression of DENND1B. Graphical abstract. (**A**) Graphical diagram of circNFIC promoted the expression of DENND1B and regulated inflammation and apoptosis by sponging miR-30e-3p. (**B**) The mRNA expression levels of DENND1B could be regulated by circNFIC. (**C**,**D**) The protein expression levels of DENND1B could be regulated by circNFIC. (**E**,**F**) The circNFIC also could regulate the mRNA expression levels of DENND1B. (**G**) CircNFIC could partly offset the suppression on DENND1B of miR-30e-3p. (**H**) There is no significant difference between miR-30e-3p alone group and co-transfected with overexpression vectors and miR-30e-3p. (**I**,**J**) Inhibitor of miR-30e-3p could rescue the down-regulation on DENND1B of knockdown of circNFIC. (**K**) Inhibitor of miR-30e-3p could rescue the down-regulation on DENND1B of knockdown of circNFIC in HD11 exposed to LPS. Data are presented as mean ± SEM (n ≥ 3 biologically independent samples). * *p* < 0.05; ** *p* < 0.01; *** *p* < 0.001; ns: no significance (Student’s *t*-test).

So far, based on the above interesting findings, we hypothesized that circNFIC plays a role through the circRNA-miRNA-mRNA network; that is, circNFIC binds miR-30e-3p competitively, releasing the binding of miR-30e-3p to DENND1B; therefore, the expression of DENND1B was regulated to promote inflammation and apoptosis (Figure 4A). To verify the regulation relationship between circNFIC and DENND1B, the overexpression vector and siRNA of circNFIC were transfected into HD11 to detect the mRNA and protein expression levels. Overexpression of circNFIC induced a significant up-regulation both of mRNA and protein levels of DENND1B (Figure 4B,C). Conversely, both mRNA and protein levels of DENND1B were notably reduced, as caused by the knockdown of circNFIC (Figure 4B,D). Furthermore, overexpression of circNFIC also promoted the mRNA expression of DENND1B in HD11 exposed to LPS (Figure 4E); on the other hand, knockdown of circNFIC suppressed the levels of DENND1B significantly with the LPS stimulation (Figure 4F). Three different volume overexpression vectors of circNFIC were co-transfected with miR-30e-3p to confirm that circNFIC regulated the expression of DENND1B by acting as a sponge of miR-30e-3p. Subsequently, the qRT-PCR assay was conducted to assess the mRNA expression levels of DENND1B (Figure 4G). After the expression of DENND1B was suppressed by miR-30e-3p, the greater the volume of circNFIC we transfected, the more DENND1B was expressed, compared with the control group (Figure 4H). In addition, the overexpression vectors or NC vectors were co-transfected with miR-30e-3p inhibitor or inhibitor NC. We then found that both circNFIC and miR-30e-3p inhibitors could promote the expression of DENND1B significantly alone; furthermore, there was no significant difference between the miR-30e-3p alone group and those co-transfected with overexpression vectors and miR-30e-3p (Figure 4H), which suggested the regulation of circNFIC to DENND1B was no longer efficacious when miR-30e-3p was suppressed. Besides, qRT-PCR and western blot were conducted to evaluate the mRNA and protein expression levels of DENND1B. Interestingly, miR-30e-3p inhibitor reversed the suppression of the knockdown of circNFIC to DENND1B (Figure 4I,J). Furthermore, in the parallel experiment in which HD11 was exposed to LPS, we found that the miR-30e-3p inhibitor could also reverse the suppression of the knockdown of circNFIC to DENND1B during LPS stimulation. 

On the whole, these results validated that circNFIC-influenced DENND1B was achieved through intermediate miR-30e-3p, to the extent that circNFIC no longer regulated DENND1B when the miR-30e-3p had a low expression.

### 3.5. DENND1B-Induced Inflammation and Apoptosis in HD11

**Figure 5:**
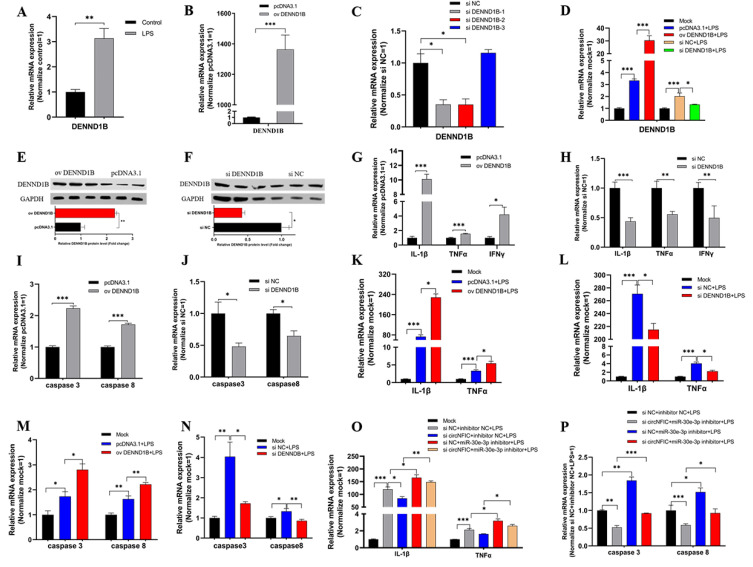
DENND1B induced inflammation and apoptosis in HD11. (**A**) The mRNA expression of DENND1B was induced by LPS stimulation. (**B**) The efficiency of overexpression and knockdown of DENND1B in HD11. (**C**,**D**) The efficiency of overexpression and knockdown of DENND1B in HD11 exposed to LPS. (**E**,**F**) The protein expression levels after overexpression and knockdown of DENND1B. (**G**,**H**) DENND1B induced the mRNA expression levels of pro-inflammatory factors (IL-1β, TNFα, and IFNγ). (**I**,**J**) DENND1B promoted the expression levels of pro-apoptosis maker genes (caspase 3 and caspase 8). (**K**,**L**) The expression of IL-1β, TNFα, and IFNγ could also be regulated by DENND1B in HD11 exposed to LPS. (**M**,**N**) DENND1B also could up-regulate the mRNA expression of pro-inflammatory factors and pro-apoptosis maker genes in HD11 exposed to HD11. (**O**,**P**) Inhibitor of miR-30e-3p could rescue the down-regulation on pro-inflammatory factors and pro-apoptosis maker genes of knockdown of circNFIC in HD11 exposed to LPS. Data were presented as mean ± SEM (n ≥ 3 biologically independent samples). * *p* < 0.05; ** *p* < 0.01; *** *p* < 0.001 (Student’s *t*-test).

After confirmation of the relationship among circNFIC, miR-30e-3p, and DENND1B, we tried to verify the functional role DENND1B plays in cell inflammation and apoptosis. Firstly, the qRT-PCR was conducted to assess the mRNA expression of DENND1B after HD11 was stimulated by LPS, and we found the expression significantly up-regulated (Figure 5A). For further research, the overexpression vector and siRNA were designed and constructed, and were transfected into HD11, both exposed to LPS and not exposed, to detect the efficiency of overexpression and knockdown of DENND1B, respectively (Figure 5B,D). Similarly, we assessed the protein expression levels after overexpression and knockdown of DENND1B (Figure 5E,F). Further, overexpressed DENND1B could dramatically promote the expression of IL-1β, TNFα, and IFNγ (Figure 5G). On the contrary, knockdown of DENND1B significantly suppressed the expression of IL-1β, TNFα, and IFNγ (Figure 5H). The expression of caspase 3 and caspase 8 was notably up-regulated or down-regulated after overexpression and knockdown of DENND1B, respectively (Figure 5I,J). In addition, we conducted similar experiments in HD11 exposed to LPS. There was no doubt that overexpressed DENND1B also could significantly promote the expression of IL-1β, TNFα, and IFNγ, as well as caspase 3 and caspase 8, under LPS stimulation (Figure 5K,M). Conversely, knockdown of DENND1B partly suppressed the effect of LPS stimulation on promoting the expression of IL-1β, TNFα, and IFNγ as well as caspase 3 and caspase 8 (Figure 5L,N). To verify that the circNFIC sponges miR-30e-3p, to affect the regulation of DENND1B to cell inflammation and apoptosis, described in this study, we co-transfected with siRNA of circNFIC and miR-30e-3p inhibitor into HD11 exposed to LPS. We found that siRNA of circNFIC mediated a significant reduction of pro-inflammation factors (IL-1β, TNFα, and IFNγ) and apoptosis marker genes (caspase 3 and caspase 8), which could be rescued by a miR-30e-3p inhibitor (Figure 5O,P).

Interestingly, we detected the mRNA expression levels of NFκB by qRT-PCR experiments. Furthermore, we found that circNFIC could significantly up-regulate the levels of NFκB in HD11 during LPS stimulating or not (Figure S3A,D). Conversely, miR-30e-3p suppressed the expression of NFκB notably (Figure S3E,F). Consequently, the levels of NFκB could be promoted significantly by DENND1B (Figure S3G,H). Taken together, we infer that circNFIC might balance inflammation and apoptosis by the miR-30e-3p/DENND1B axis through the NFκB pathway.

## 4. Discussion

*Eimeria tenella* is a specific intracellular parasitic protozoa [45], that can parasitize chicken intestinal epithelial cells and cause severe epidemic chicken coccidiosis [44]. Coccidiosis can cause intestinal inflammation and bleeding in chickens [44], resulting in decreased appetite and absorptive capacity, decreased nutrient intake, and could even cause the host’s death in severe cases [4]. Coccidiosis causes great losses to the poultry industry [3,4]. After invasion into the host, coccidia could induce the humoral immunity and cellular immunity [6]. In addition, cellular immunity mainly includes the cellular functions of T cells, macrophages and natural killer cells [46]. Among these, macrophages can exert immune regulation in two manners. On the one hand, it can directly phagocytize the coccidia sporozoites to prevent their continued reproduction and invasion. On the other hand, it can also play a key role by secreting inflammatory factors and enhancing the antigen presentation [47]. In this paper, chicken macrophage cell lines (HD11) were used as the research object, and the inflammation model, induced by LPS stimulation, was used to simulate the infection caused by coccidia invasion.

Lipopolysaccharide (LPS) is the main component of the cytoderm of gram-negative bacterium, including conservative lipid A and polysaccharides [48]. Firstly, LPS is bound with LPS binding protein (LBP); then, the complexes are recognized and bound by membrane clusters of differentiation 14 (mCD14), and toll-like receptors (TLRs) transmitted the signal into cells [49]. TLRs can recognize specific components of bacteria, including LPS, pro-inflammatory factors, including IL-1β and TNF-α, which are induced and secreted in abundance [49]. TNF-α was a multifunction molecule, which not only promoted the progression of inflammation but also had an effect on caspase cascade reaction then significantly induced exogenous apoptosis [50]. Caspase families are conserved and are involved in cell death and inflammation and are divided into initiator (including caspases 8, 9, and 10) and effector (including caspases 3, 6, and 7) functionalities [51]. Besides, the TNF receptor assembling the death-inducing signaling complex (DISC) is the key step for activating caspase 8 and caspase 10 [52]. After that, the zymogens of apoptotic effector caspases 3 gain proteolytic activity through caspase 8 [53]. Consequently, reactive oxygen species (ROS) were produced in abundance.

Many diseases, including obesity, Alzheimer’s disease, and renal cell carcinoma, are associated with inflammation [54,55,56]. Correspondingly, increasing evidence has asserted that circular RNA is relevant to inflammation-related diseases [54] and could be used as novel diagnostic biomarkers [15]. Furthermore, we have demonstrated that circNFIC not only induced inflammation but also promoted apoptosis in macrophages exposed to LPS stimulation or not (Figure 2). There are many ways in which circNFIC functions in the cell. The circE7 regulated the transformed growth of cervical carcinoma cells by translating to produce E7 oncoprotein [57]. Furthermore, circFOXO3 can interact with ID-1, E2F1, FAK, and HIF1α and increase cellular senescence [58]. Compared to the description above, more circular RNAs were reported to function as a microRNA sponge [19,21,59]. To explore that mechanism, the RNA pull-down, dual-luciferase reporter assay, and the RNA FISH assay were carried out, and many qRT-PCRs were performed. Lastly, we demonstrated that circNFIC regulated the expression of DENND1B, aggravating inflammation and apoptosis by sponging miR-30e-3p in macrophages either exposed to LPS or not.

The nuclear factor I (NFI) family, or CCAAT box-binding transcription factor (CTF) family [60], included four members—NFIA, NFIB, NFIC, and NFIX [61]. A study suggested that NFIC could balance adipogenic and osteogenic differentiation through the Wnt signaling pathway, which was regarded as a novel target for controlling metabolic disorders, such as obesity [62]. In addition, NFIC was reported to suppress the expression of pro-inflammatory factors, including IL-6, IL-8, and TNF-α, stimulated by LPS at a dose of 1 μg/mL in human stem cells from the apical papilla [41]. Interestingly, this is the opposite of the function of circNFIC. Furthermore, we assessed the expression of NFIC mRNA after overexpression or knockdown of circNFIC, and we found overexpressed circNFIC could dramatically down-regulate the levels of NFIC. On the contrary, the knockdown of circNFIC significantly induced up-regulation of NFIC in HD11 (Figure S2). Therefore, there is some effect of circNFIC on NFIC. We supposed that circNFIC might interact with RNA binding protein (RBP) to suppress the transcription of NFIC. In addition, many circular RNAs were reported to translate through cap-independent mechanisms [57]. The possibility existed that circNFIC translated and competed for elements of transcription or translation. Thus, circNFIC could suppress the expression of NFIC to regulate inflammation through another pathway.

Tumour necrotic factor-α (TNFα) is a pleiotropic pro-inflammatory cytokine, which can be produced by various cell types, such as T cells, macrophages, and eosinophils [63]. TNFα is involved in the inflammatory response to bacterial, viral, and parasitic infections by activating macrophages to produce carbonic oxide to suppress the mitochondria productivity, causing the parasites to die from energy drain [11]. Interferon-γ (IFNγ), which is a protein that could play a role in anti-viral and anti-foreign effects, can be produced by NK cells and macrophages, and it promotes the expression of MHC Ι and MHC ΙΙ and enhances the toxicity of NK cells [11]. There are differences between TNFα and IFNγ, which might be the reason that IFNγ did not show exactly the same trend as TNFα and IL-1β during the exposure of HD11 to LPS (Figure 2, Figure 3 and Figure 5), which is different from normal. This is similar to how IFNγ significantly inhibits uracil incorporation in retinal muller glial to inhibit T. gondii replication [64], but TNFα does not, which is a good example to show the difference between TNFα and IFNγ.

Nuclear factor-kappa B (NFκB) is a family of dimeric transcription factors [65]. As a key regulator of immune responses, inflammation, and cancer, NFκB has been a popular factor among scientists for the past decades [66]. TLR family can recognize specific microbial patterns, such as LPS, then recruit MYD88 to mediate the activation of the NFκB pathway [67]. Moreover, pro-inflammatory cytokines, such as TNFα and IL-1, are also involved in the NFκB signal activation [68]. At the same time, caspase 8 is activated to promote apoptosis [67]. For now, we noticed that NFκB signal could be activated by multiple factors: microorganisms, such as LPS, and inflammatory factors, such as TNFα and IL-1. This period is accompanied by apoptosis. Further, the qRT-PCR revealed that circNFIC, miR-30e-3p, and DENND1B could all regulate the expression levels of NFκB, which might mean that DENND1B balanced inflammation and apoptosis by acting as a potential regulator of NFκB. Both circNFIC and miR-30e-3p play roles on inflammation and apoptosis by influencing the expression of DENND1B, and, consequently, activating the NFκB pathway to regulate the secretion of cytokines in HD11.

## 5. Conclusions

In this study, we explored the roles that circNFIC, miR-30e-3p, and DENND1B play in cell inflammation and apoptosis, and validated circNFIC-regulated DENND1B expression, which exerted pro-inflammatory and pro-apoptosis effects when sponging miR-30e-3p. Consistently, the effect of knockdown of circNFIC in suppressing the expression of pro-inflammatory and pro-apoptosis could be reversed by a miR-30e-3p inhibitor. These data demonstrated that circNFIC aggravated inflammation and apoptosis via the miR-30e-3p /DENND1B pathway in HD11.

## Data Availability

The data presented in this study are available on request from the corresponding author.

## Supplementary Materials

The following are available online at https://www.mdpi.com/article/10.3390/genes12111829/s1, Figure S1: The inflammatory model was constructed inducing by LPS, Figure S2: The mRNA of NFIC could be suppressed by circNFIC, Figure S3: The circNFIC balanced the expression of NFκB by miR-30e-3p/DENND1B axis, Figure S4: RNA-FISH revealed the localization of circNFIC and miR-30e-3p. Table S1: The primers used in this study, Table S2: RNA oligonucleotides sequence information.
