# Supplementary material for: CircNFIC Balances Inflammation and Apoptosis by Sponging miR-30e-3p and Regulating DENND1B Expression
Source: Genes (Basel). 2021 Nov 19;12(11):1829. doi: 10.3390/genes12111829 (PMC8622209; doi:10.3390/genes12111829)
Supplement: Supplementary file 1 [file genes-12-01829-s001.zip › genes-1428526-supplementary (1)-done.pdf]

genes

MDPI

Supplementary Material

1. Supplementary Figures

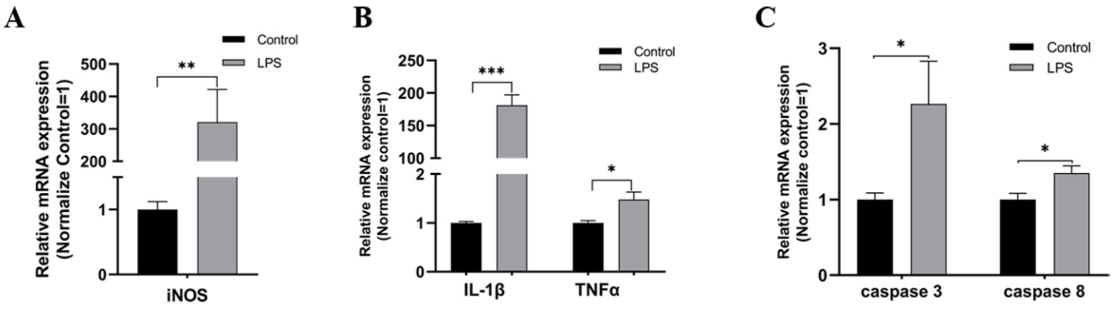

Supplementary Figure S1. The inflammatory model was constructed inducing by LPS. (a) The mRNA expression of iNOS was significantly promoted by LPS. (b) The mRNA expression of IL-1β and TNF-α were significantly promoted by LPS. (c) The mRNA expression of caspase 3 and caspase 8 were significantly promoted by LPS.

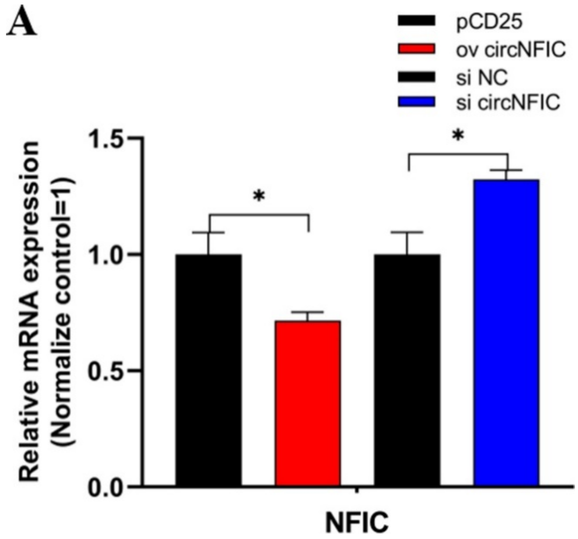

Supplementary Figure S2. The mRNA of NFIC could be suppressed by circNFIC. (a) The mRNA expression of NFIC could be suppressed by circNFIC.

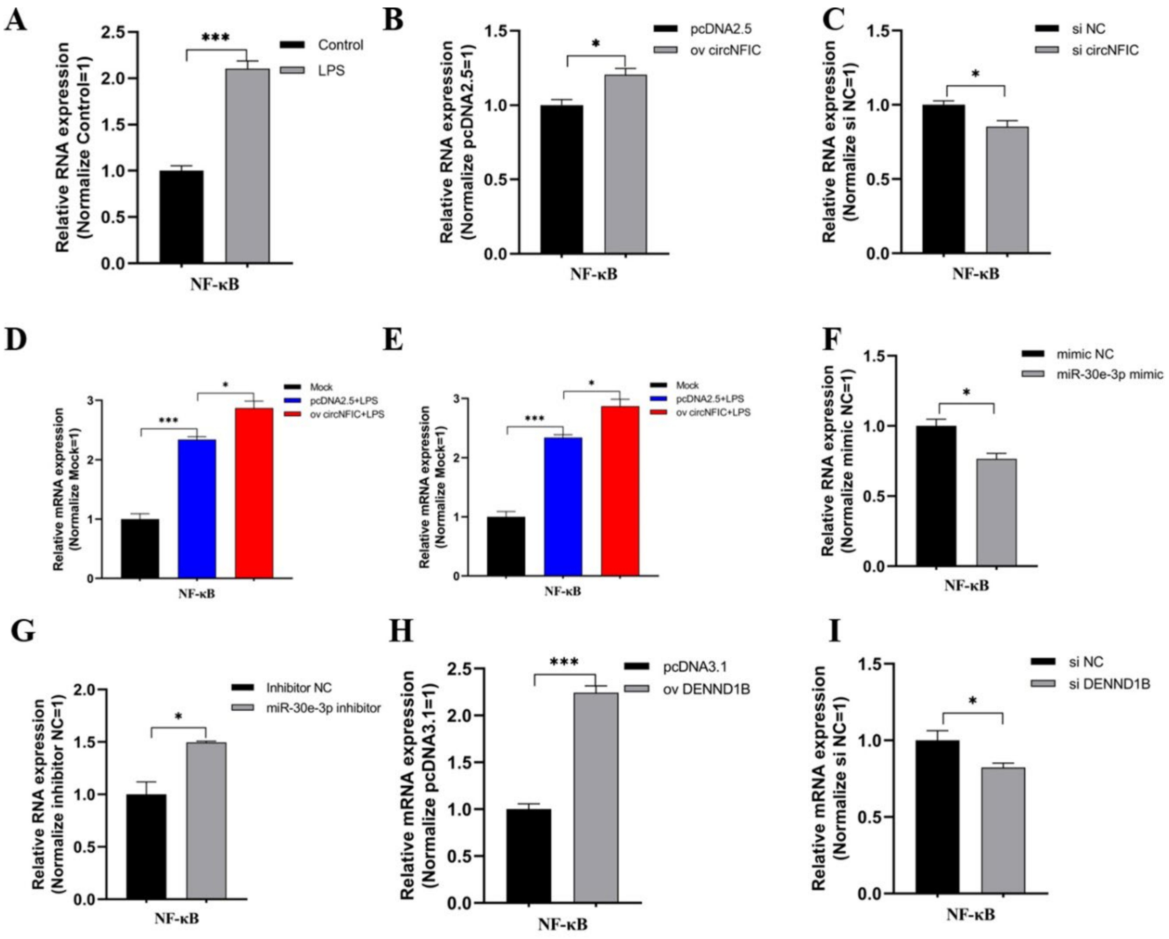

Supplementary Figure S3. The circNFIC balanced the expression of NFκB by miR-30e-3p/DENND1B axis. (a) The mRNA expression of NFκB was significantly promoted by LPS. (b, c) The expression levels of NFκB with overexpression and knockdown of circNFIC in HD11. (d, e) The expression levels of NFκB with overexpression and knockdown of miR-30e-3p in HD11. (f, g) The expression levels of NFκB with overexpression and knockdown of DENND1B in HD11. (h, j) The expression levels of NFκB could be regulated by circNFIC, miR-30e-3p, and DENND1B in HD11 exposing to LPS.

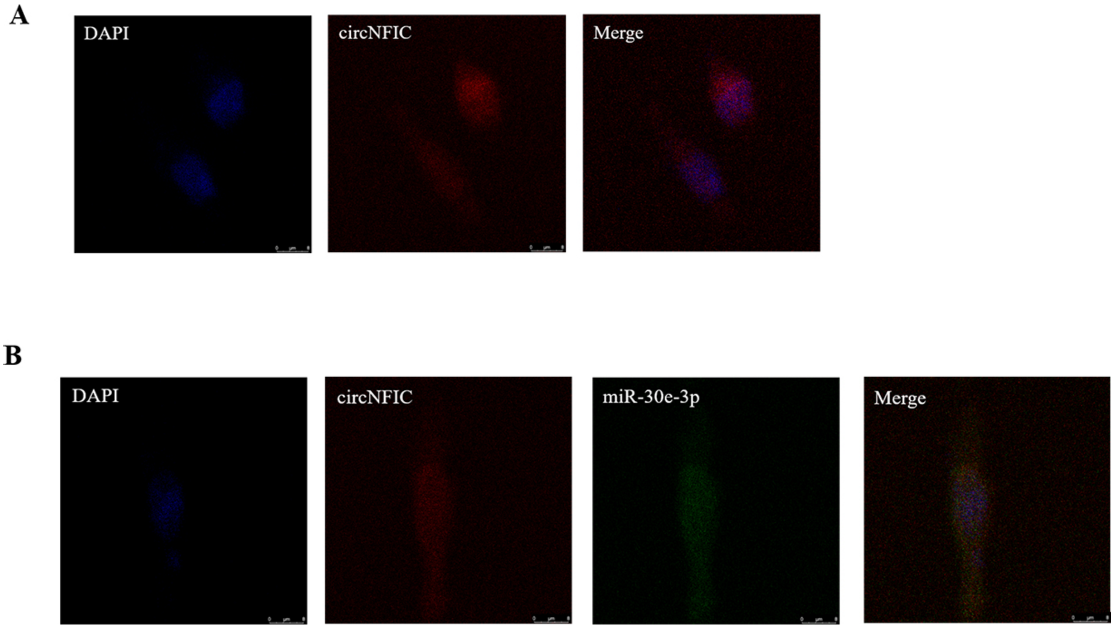

Supplementary Figure S4. RNA-FISH revealed the localization of circNFIC and miR-30e-3p. (a) RNA-FISH revealed the localization of circNFIC. (b) RNA-FISH revealed the co-localization of circNFIC and miR-30e-3p. The circACSL1 probes were labeled with CY3 (red), miR-30e-3p probes were labeled with FAM. Nuclei were stained with DAPI (blue).

2. *Supplementary table*

Supplementary Table S1. The primers used in this study.

Primer name	Fragment sequence (5'-3')	Application
NFIC-F	ACCACATCAGCGTCTCCGTCAA	PCR
NFIC-R	GCGTTCGGGACACCTGGAT	PCR
circNFIC-qF	ACGGCCTCCACCTATTTCCC	qPCR
circNFIC-qR	TGTCCAGTTGCGGCTGCTACT	qPCR
DENND1B-qF	ACCAGCACCTCAATAACCA	qPCR
DENND1B-qR	GCTCAGCGTGTCCAATATC	qPCR
IL-1β-qF	TCGGGTTGGTTGGTGATG	qPCR
IL-1β-qR	TGGGCATCAAGGGCTACA	qPCR
TNF-α-qF	TTCTATGACCGCCCAGTT	qPCR
TNF-α-qR	CAGAGCATCAACGCAAAA	qPCR
IFNγ-qF	GACATCCTTCAGCATCTCTTCA	qPCR
IFNγ-qR	AGGCGCTGTAATCGTTGTCT	qPCR
iNOS-qF	CCGTTGAAAGGACACGCAGT	qPCR
iNOS-qR	TATTTGAATACCCGCCAGAG	qPCR
caspase 3-qF	TGGCCCTCTTGAACTGAAAG	qPCR
caspase 3-qR	TCCACTGTCTGCTTCAATACC	qPCR
caspase 8-qF	CCCTGAAGACAGTGCCATTT	qPCR
caspase 8-qR	GGGTCGGCTGGTCATTTTAT	qPCR
NFκB-qF	AGGATGAGAATGGTGGTATG	qPCR

NFκB-qR	CAGATTTCCGACGTAGTTGT	qPCR
NFIC-qF	TACCTGCCCACGCAGATGCTG	qPCR
NFIC-qR	CGTCCCTCCCTCCGAAGTTGT	qPCR
β-actin-qF	TTGTTGACAATGGCTCCGGT	qPCR
β-actin-qR	AACCATCACACCCTGATGTCT	qPCR

Supplementary Table S2. RNA oligonucleotides sequence information.

Sequence name	Fragment sequence (5'-3')	Application
si-circNFIC_001	GACCGGAGCAAACGGCACA	circNFIC knockdown
si-circNFIC_002	ACCGGAGCAAACGGCACAA	circNFIC knockdown
si-DENND1B_001	CAAAGAGTCTGGACTTCTT	DENND1B knockdown
si-DENND1B_002	GCATCGTTCCAGTACTACA	DENND1B knockdown
si-DENND1B_003	GGATGTACACCGTGAAGAA	DENND1B knockdown
miR-30e-3p mimic	UUUCAGUCGGAUGUUUACAGC	miR-30e-3p overexpression
miR-30e-3p inhibitor	GCUGUAAACAUCCGACUGAAA	miR-30e-3p knockdown
